# Continuous high-dose infusion of doripenem in a pneumonia patient infected by carbapenem-resistant *Pseudomonas aeruginosa*: a case report

**DOI:** 10.1186/s40780-019-0144-4

**Published:** 2019-07-08

**Authors:** Kazutaka Oda, Hidenobu Kamohara, Tomomi Katanoda, Yumi Hashiguchi, Koji Iwamura, Kisato Nosaka, Hirofumi Jono, Hideyuki Saito

**Affiliations:** 1 Department of Pharmacy, Kumamoto University Hospital, 1-1-1, Honjo, Chuo-ku, Kumamoto, Japan; 2 Department of Infection Control, Kumamoto University Hospital, 1-1-1, Honjo, Chuo-ku, Kumamoto, Japan; 3 Department of Critical Care Medicine, Kumamoto University Hospital, 1-1-1, Honjo, 860-8556 Chuo-ku, Kumamoto, Japan

**Keywords:** Doripenem, Continuous infusion, High-dose, Therapeutic drug monitoring, Continuous renal replacement therapy, Acute kidney injury

## Abstract

**Background:**

Despite the high mortality of patients with sepsis and carbapenem-resistant bacteria infection, appropriate antimicrobial therapies are yet to be established. Here, we have reported the case of a patient with pneumonia that subsequently developed by carbapenem-resistant *Pseudomonas aeruginosa* infection and was treated with a continuous high-dose infusion of doripenem.

**Case presentation:**

We started a continuous intravenous infusion of doripenem 3 g/day although the 59-year-old woman (body weight, 45 kg) had developed septic acute kidney injury, followed by continuous renal replacement therapy (the effluent flow rate was 650 mL/h). The minimum inhibitory concentration (MIC) of doripenem was 8 mg/L. The concentration of unbound doripenem in the serum was measured by using high-performance liquid chromatography. Twenty hours after the initial dose, the patient’s serum level of doripenem was 47.8 μg/mL; the level decreased to 33.6 μg/mL at 111 h after initial dosing. The unbound doripenem concentration in the serum was maintained four times above the MIC throughout the treatment. After the completion of 11 days of dosing, the patient was discharged from the intensive care unit. During the treatment period, the MIC remained at 8 mg/L.

**Conclusions:**

A continuous high-dose infusion of doripenem is a potentially efficient strategy for the treatment of antimicrobial-resistant bacteria. Moreover, therapeutic drug monitoring may be useful for patients displaying variable pharmacokinetics, because the MIC is generally high in resistant bacteria.

## Background

The mortality rate of patients with sepsis is reported to be greater than 10% [1]; therefore, appropriate antimicrobial therapies must be applied to ensure a successful cure. Owing to the broad-spectrum antibacterial activity of carbapenems, they are often used for the treatment of patients with sepsis; however, carbapenem-resistant bacteria have emerged as a major concern for medical practitioners [2]. A mortality rate of up to 21.4% has been reported for patients with sepsis and carbapenem-resistant *Pseudomonas aeruginosa* infection [2]. Despite the available treatment options for *P. aeruginosa*, monotherapy with aminoglycosides was reported to result in a higher mortality rate than that of combination therapies [2]. Although colistin is a potential antibiotic treatment for *P. aeruginosa*, adverse reactions, such as nephrotoxicity and neurotoxicity, are of great concern [3]. Therefore, appropriate antimicrobial therapies must urgently be established for patients with sepsis and carbapenem-resistant *P. aeruginosa* infection.

An adult female patient developed pneumonia and, subsequently, infection by a *P. aeruginosa* strain that was resistant to both fluoroquinolones and carbapenems. In the 14 days after diagnosis, she was administered tobramycin (a type of aminoglycoside), which has a minimum inhibitory concentration (MIC) of 1 mg/L. However, the clinical effect was poor: the patient developed respiratory failure and acute kidney injury (AKI), and she was then transferred to our intensive care unit (ICU). The MIC of doripenem (a type of carbapenem [4]) was at a level at which the strain was resistant (8 mg/L); some reports have shown that meropenem (a carbapenem, with an MIC of > 8 mg/L in this case) was successfully cured at a high dose by keeping the serum meropenem concentration above the MIC against *P. aeruginosa* [5, 6].

In this study, we have reported a case of a successful cure by the application of a continuous high-dose infusion of doripenem in addition to tobramycin administration.

### Case presentation

**Fig. 1:**
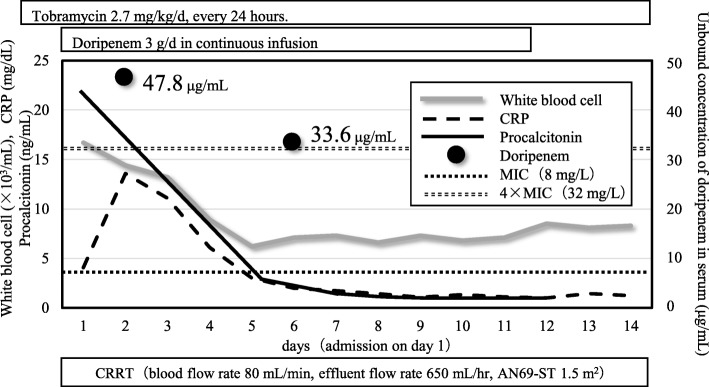
Time course of values for doripenem and other laboratory tests. The concentration of unbound doripenem in serum was 47.8 μg/mL at 20 h after dosing was started, and decreased to 33.6 μg/mL at 111 h. All biomarkers that acted as an indicator of infection were included in the calculations for the respective normal ranges. The serum concentration of tobramycin was measured on Day 1 before the admission of the patient to the ICU (peak, 16.5 μg/mL; trough, 4.3 μg/mL); the tobramycin dose was then decreased from 5.3 mg/kg/day to 2.7 mg/kg/day. CRP, C-reactive protein; CRRT, continuous renal replacement therapy; AN69-ST, a dialyzer membrane made by polyacrylonitrile (surface-treated)

Sixty-three days before starting therapy for this infection, a 59-year-old female patient had been discharged on Day 22 post-surgery in our ICU following a mitral valve replacement. However, at 41 days after discharge, she developed pneumonia due to infection by *P. aeruginosa*, and thus returned to the ICU, where she had to be kept on mechanical ventilation (owing to respiratory failure) and continuous renal replacement therapy (CRRT; owing to AKI). A dosing strategy for doripenem was calculated to determine the continuous infusion to achieve a certain target serum concentration of the unbound drug, which was set to 32 μg/mL, i.e., four times higher than the actual MIC (8 mg/L) [7]. Here, if the maximal licensed dose in Japan (3 g/day) would be delivered by continuous intravenous infusion (1 g/80 mL of normal saline/8 h; 10 mL/h, every 8 h), the target concentration could only be achieved when the doripenem total clearance (CLtot) was < 3.6 L/h. However, previous reports have indicated a doripenem clearance of 2.7–5.9 L/h by the body (CL_BODY_) [8–12]; doripenem clearance by CRRT (CL_CRRT_) in this case was calculated to be 0.6 L/h based on the effluent flow rate of CRRT [13]. Therefore, the probability that the patient’s CLtot was < 3.6 L/h was low. Furthermore, Monte Carlo simulation computed by R (ver. 3.5.3, https://www.r-project.org/) estimated that there was a 7.6% probability of achieving 32 μg/mL of unbound doripenem by using the population pharmacokinetic model for doripenem reported by Roberts et al. [11]. Therefore, therapeutic drug monitoring (TDM) was applied. The time course of the values for the concentration of unbound doripenem in the serum and the values from other laboratory tests during the patient’s stay at the ICU are presented in Fig. 1. The samples were prepared by ultrafiltration using a Nanosep Omega 10 K and the concentrations of unbound doripenem in the serum were quantified by high-performance liquid chromatography [14]. The concentration of unbound doripenem was 47.8 μg/mL at 20 h after the dose administration started. The concentration decreased to 33.6 μg/mL at 111 h, although these concentrations were maintained at levels that were four times higher than the MIC. Continuous infusion of doripenem was performed for 11 days, after which the patient was discharged from the ICU. After the end of the continuous infusion of doripenem, the MIC against *P. aeruginosa* detected in her sputum was 8 mg/L, which was not elevated.

## Discussion and conclusions

In this case study, we have reported that a continuous high-dose infusion of doripenem provided a successful cure for a patient who had developed pneumonia and doripenem-resistant *P. aeruginosa* infection.

Although colistin may be used as an antibiotic against carbapenem-resistant *P. aeruginosa*, it is reported to cause adverse reactions, such as nephrotoxicity and neurotoxicity [3]. Therefore, colistin use is only recommended in when other antibiotics cannot be used [3]. To the best of our knowledge, only a few reports are available on the dose-dependent adverse reactions of doripenem; therefore, high-dose doripenem administration may be safer than the normal dosage of colistin. To date, the pharmacokinetic/pharmacodynamic (PK/PD) target of beta lactams for successful bactericidal activity has been the time for which the unbound drug concentration above the MIC (%*f*T > MIC), with a target value of 40% for carbapenems [15]. The PK/PD target has been now been updated to suggest that the concentration of unbound drug should be more than four times higher than the MIC (%*f*T > 4 × MIC) for the maximal therapeutic effect. Therefore, a target value of 60% or 100% has been advocated [7]. As the unbound fraction of doripenem has been reported to be 91.1% [4], we aimed to maintain a concentration of unbound doripenem more than four times higher than the MIC by using a continuous infusion (32 mg/L).

Previous reports have suggested a variety of dosing strategies for doripenem in patients with CRRT [8–12]. However, only a small amount of information is available for doripenem-resistant *P. aeruginosa*. In this case, although we started from the maximal licensed dose (3 g/day), the probability of achieving the target concentration appeared to be low [8–13]; therefore, we applied TDM.

TDM was conducted by using serum samples taken for other laboratory tests; CLtot was calculated to be 2.4 L/h at the time when the concentration of unbound doripenem was 47.8 μg/mL in the serum. The equation is presented below.

$$ " CLtot= dose\kern0.5em \left(125\kern0.5em mg/h\right)\times unbound\ fraction\kern0.5em (0.911)/ unbound\ concentration\;\left(47.8\; mg/L\right)" $$

The CL_CRRT_ was calculated to be 0.6 L/h from the effluent flow rate of CRRT. The equation was described below.

$$ "{CL}_{CRRT}= effluent\ flow\ rate\;\left(0.65\;L/h\right)\times unbound\ fraction\;(0.911)" $$

Therefore, CL_BODY_ was calculated to be 1.8L/h (*CL*_*BODY*_ = *CLtot* – *CL*_*CRRT*_). Because CLtot and CL_BODY_ were smaller than the assumption and %*f*T > 4 × MIC was 100%, further optimization of the dose was not necessary. In contrast, kidney function improved as demonstrated by the alterations in both the urine output (first day, 56 mL/day; 6 days later, 498 mL/day) and serum creatinine levels (first day, 0.97 mg/dL; 6 days later, 0.79 mg/dL) during therapy. The improved kidney function might have resulted in the decreased in the concentration of unbound doripenem in the serum to 33.6 μg/mL, when CLtot, CL_CRRT_, CL_BODY_, and %*f*T > 4 × MIC was calculated to be 3.4 L/h, 0.6 L/h, 2.8 L/h, and 100%, respectively. Importantly, because the initial serum creatinine level was not abnormally high, it was difficult to estimate kidney function precisely in a patient with CRRT. Moreover, the concentration of unbound doripenem in the serum 6 days after the initial dose decreased to a level that was almost near the lower boundary of the target concentration, indicating that CLtot and CL_BODY_ varied during therapy. Collectively, the application of TDM in combination with the continuous infusion of doripenem may be useful in patients with CRRT.

This study had several limitations. First, the safety of a continuous high-dose infusion of doripenem has not been confirmed. Thus, careful intervention, such as TDM, is required. Second, the concomitant use of tobramycin may play a key role in the provision of a successful cure and the prevention of emerging resistant *P. aeruginosa* strains rather than the administration of a continuous high-dose infusion of doripenem [16]. In contrast, it has been reported that the concomitant use of aminoglycosides with carbapenems can result in negative therapeutic effects [16]. Therefore, further studies of a continuous high-dose infusion of doripenem should be performed.

In conclusion, this was the first case in which a continuous high-dose infusion of doripenem provided a successful cure against pneumonia caused by doripenem-resistant *P. aeruginosa*. Moreover, TDM may be useful for patients with variable pharmacokinetics because the MIC is generally high in resistant bacteria.

## Data Availability

The datasets used and/or analyzed during the current study are available from the corresponding author upon reasonable request.

## Abbreviations

%*f*T > 4 × MIC: Time of unbound drug concentration above 4 × MIC
%*f*T > MIC: Time of unbound drug concentration above MIC
AKI: Acute kidney injury
AN69-ST: A dialyzer membrane made by polyacrylonitrile (surface-treated)
CL_BODY_: Clearance by body
CL_CRRT_: Clearance by continuous renal replacement therapy
CLtot: Total clearance
CRRT: Continuous renal replacement therapy
ICU: Intensive care unit
MIC: Minimum inhibitory concentration
PK/PD: Pharmacokinetic/pharmacodynamic
TDM: Therapeutic drug monitoring
